# Association Between County-Level Social Vulnerability and Vaccine-Related Attitudes and Hesitancy Toward COVID-19 Vaccination in the United States

**DOI:** 10.3390/vaccines12121368

**Published:** 2024-12-03

**Authors:** Yun Kim, Ronaldo Iachan, John Boyle, Yangyang Deng

**Affiliations:** ICF International, Rockville, MD 20850, USA

**Keywords:** health disparities, vaccine hesitancy, attitudes, social vulnerability index (SVI), COVID-19

## Abstract

Background/Objectives: Understanding attitudes and behaviors related to vaccination is critical for enhancing COVID-19 vaccination acceptance and reducing disparities in vaccination coverage. This study examines disparities in vaccine-related attitudes and COVID-19 vaccine hesitancy in the United States in relation to community-level social vulnerability. Methods: This study analyzed cross-sectional national surveys conducted repeatedly between July 2020 and August 2021 (*n* = 6716). We assessed the association between county-level social vulnerability and general vaccine-related attitudes, as well as COVID-19 vaccine hesitancy. We developed Poisson models with robust variance estimation. The analysis also included the association of county social vulnerability with parental COVID-19 vaccine hesitancy. Results: Living in counties with high Socioeconomic Status vulnerability was associated with less vaccine support (adjusted Prevalence Ratio (aPR) 1.10; 95% CI 1.05–1.14) and residing in counties with high Household Characteristics vulnerability was associated with higher likelihood of COVID-19 vaccine hesitancy (aPR 1.13; 95% CI 1.07–1.20). In contrast, high vulnerability in the Racial and Ethnic Minority was associated with more positive attitudes toward vaccines (aPR 0.91; 95% CI 0.88–0.94) and lower COVID-19 vaccine hesitancy for both themselves (aPR 0.81; 95% CI 0.76–0.87) and children (aPR 0.84; 95% CI 0.75–0.94), after adjusting for sociodemographic factors. Conclusions: Our study highlights the importance of addressing vulnerabilities related to socioeconomic status and household characteristics to reduce disparities in vaccine perceptions and hesitancy in socially vulnerable populations. The findings provide evidence for targeted public health interventions at the community level. They also demonstrate that the relationship between social vulnerability and vaccine attitudes varies across different vulnerability components.

## 1. Introduction

Vaccination plays a vital role in preventing COVID-19 infection and reducing risk of severe illness, hospitalization, and death for both adults and children [1,2,3]. As the SARS-CoV-2 virus continues to evolve, receiving updated COVID-19 vaccines remains essential. By 19 April 2021, COVID-19 vaccines were authorized for all adults in the United States. Vaccination was later authorized for children 12–15 years old on 10 May 2021, for children 5–11 years old on 29 October 2021, and for children 6 months to 4 years old on 17 June 2022. As vaccine effectiveness began to wane, booster shots were recommended for all adults by November 2021. The rollout of bivalent boosters or updated vaccines, designed to target both the original strain and Omicron variants, began on 1 September 2022. As of December 2021, approximately 85% of adults aged 18 and older in the U.S. had received at least one dose of a COVID-19 vaccine [4], although coverage for booster or updated vaccines remained relatively low: by May 2024, only 22.5% of adults and 14.4% of children had received the updated 2023-24 COVID-19 vaccine since September 2023 [5].

Vaccine hesitancy, defined as the delay in acceptance or refusal of vaccines despite their availability, has been identified as one of the most significant barriers to enhancing COVID-19 vaccination coverage in the U.S. [6]. This phenomenon, recognized by the World Health Organization as one of the top ten threats to global health, has become particularly salient during the COVID-19 pandemic [7]. The Health Belief Model provides a framework for understanding vaccine hesitancy and acceptance. According to this model, attitudes and beliefs towards vaccines have been suggested as crucial predictors of COVID-19 vaccination acceptance and hesitancy [8,9,10]. Individuals’ attitudes towards vaccine acceptance or refusal are shaped by the interplay of perceived benefits and risks of vaccines, as well as perceived susceptibility to and severity of diseases [11,12]. Additionally, these perceptions are influenced by both individual- and community-level social determinants, including socioeconomic factors, access to healthcare, cultural practices, and community norms [13,14].

Recent research highlights significant disparities in vaccination hesitancy or acceptance across various socioeconomic and demographic groups in the U.S. [15,16,17,18]. Studies have found that individuals from racial and ethnic minority backgrounds, those with lower levels of educational achievement, and lower household income generally exhibit higher levels of COVID-19 vaccine hesitancy [15] and lower vaccination coverage rates [16,17]. For instance, a national sample study in the U.S. found that the odds of COVID-19 vaccine hesitancy or refusal were 1.68 times greater among Black/African American compared to White people [18]. Factors contributing to these disparities include lack of trust in government and health authorities, lack of confidence in vaccine safety, concerns about side effects, religious or spiritual barriers, and poor knowledge about the disease [19,20].

The Social Vulnerability Index (SVI) is a valuable tool for quantifying the multidimensional nature of social vulnerability by incorporating data on various indicators including demographic factors and social determinants of health. The SVI, originally conceived as a measure of vulnerability to disease outbreaks and natural disasters [21], was updated in 2020 to consider 16 different indicators grouped under four broad social vulnerability domains: (a) Socioeconomic Status (SES), (b) Household Characteristics, (c) Racial and Ethnic Minority Status, and (d) Housing Type and Transportation. Federal and state governmental agencies have employed the SVI for equitable and effective resource distribution in responding to the COVID-19 pandemic, such as setting up drive-thru and community-based testing sites and allocating vaccines [22].

While disparities in COVID-19 vaccine hesitancy and refusal have been well documented, there has been limited investigation into the disparities in general vaccine-related beliefs and attitudes, which are key components and strong predictors of vaccine hesitancy, as highlighted in previous studies [8,23]. Our study provided a unique perspective by examining the general beliefs about vaccines within the context of SVI, rather than specifically about COVID-19 vaccine. This approach helps better understand the disparities in the broader public acceptance or resistance to vaccines. Consequently, our findings have wider applicability to various vaccines in potential future outbreaks. Furthermore, while individual-level sociodemographic determinants of parental COVID-19 vaccine hesitancy for children has been documented [24,25], there is a lack of studies on assessing disparities in the parental hesitancy toward pediatric COVID-19 vaccination within the context of community-level social vulnerability. To address this gap, we conducted the subgroup assessment focusing on participants with children.

Our study examined the association of the four social vulnerability domains with three measures: (1) general attitudes toward vaccines, (2) vaccine hesitancy specific to COVID-19 among adults, and (3) parental COVID-19 vaccine hesitancy for their children in the U.S. population by using the data collected between April 2020 and August 2021. By examining how social vulnerability predicts vaccine attitudes and behaviors, this study seeks to contribute to the broader discourse on health equity and inform public health strategies to improve vaccination coverage across diverse communities.

## 2. Methods

### 2.1. Participants and Data Collection

This study utilized data collected for a project examining public attitudes and behaviors during the COVID-19 pandemic. Participants were sourced from the MFour mobile panel, a national non-probability panel comprising about two million individuals across the U.S. The panel samples of *n* = 3000 adults in each wave were nationally representative along geographic and demographic factors including age, gender, race/ethnicity, education, and zip code. Data from 6716 participants were collected through seven waves between July 2020 and August 2021. The first five waves occurred monthly from July to November 2020, with additional waves conducted in April and August 2021. Panelists received survey invitations via cell phone app notifications, and non-respondents were sent up to three reminders. The sample sizes across the waves ranged from 917 to 1000 (Supplemental Table S1). We conducted poststratification to calculate survey weights and adjust the data based on U.S. adult population demographics across the following dimensions: age, sex, race and ethnicity, education, and marital status. This study was reviewed and approved by the Institutional Review Board (or Ethics Committee) of ICF for the protection of human subjects (protocol code 2020–149 approved on 25 March 2020).

### 2.2. Survey Measures

The survey aimed to assess various aspects related to vaccination and COVID-19, including general attitudes toward vaccines, perceptions of COVID-19 prevention, and trust and confidence in government agencies and other institutions. We utilized 11 items related to general vaccine or immunization beliefs (Cronbach’s alpha = 0.92), such as perceived risk (e.g., “Some vaccines are linked to long-term health problems”), perceived benefit (e.g., “Overall, vaccines are very effective”), and perceived safety (e.g., “Overall, vaccines are very safe”). Responses to these items were measured using a 4-point Likert scale: “strongly disagree”, “somewhat disagree”, “somewhat agree”, and “strongly agree”. This survey tool was employed to understand immunization beliefs, intentions, and behaviors in a previous study [26].

For survey waves conducted in 2020, when COVID-19 vaccines were not yet publicly available, we assessed behavioral intent toward COVID-19 vaccination by asking: “How likely are you to try and get the coronavirus (COVID-19) vaccine as soon as an FDA-approved one becomes available?” After the COVID-19 vaccine became available in mid-April 2021, we added a question to determine whether participants had received a COVID-19 vaccine. For unvaccinated respondents, we followed up with: “How likely are you to get a COVID-19 vaccine?”

For participants with children under 18, we assessed parental behavioral intent regarding their children’s COVID-19 vaccination using the question: “How likely are you to get the coronavirus (COVID-19) vaccine as soon as an FDA-approved one becomes available for your child?” Consistent with adult vaccination, this evolved to be a two-step question format: first, inquiring about past vaccination and then about willingness if they had not yet received it. For respondents with two or more children of different ages, these questions were asked separately for each child. Responses to COVID-19 vaccine willingness were measured using a 4-point Likert scale: “very likely”, “somewhat likely”, “not too likely”, and “not at all likely”. Additionally, we collected key demographic information, including age, gender, race and ethnicity, political affiliation, education, and household income.

### 2.3. Independent Variables

The primary independent variables were county-level social vulnerability from the CDC 2020 Social Vulnerability Index (SVI). We linked the respondents’ zip codes to the county Federal Information Processing Standard (FIPS) codes and merged the SVI data with the survey data using FIPS codes. We classified each county into low, medium, and high levels of social vulnerability according to terciles of the scores of the four vulnerability domains, across all U.S. counties [27,28]. Table 1 outlines the social factors contributing to each vulnerability domain used for ranking the counties. The 16 social factors were derived from data collected in the American Community Survey (ACS) from 2016 to 2020 [21].

### 2.4. Dependent Variables

The analysis focused on three dependent variables: (a) general attitude towards vaccines, (b) adults’ COVID-19 vaccination hesitancy, and (c) parents’ hesitancy concerning vaccinating their children against COVID-19. First, we employed Latent Class Analysis (LCA) to uncover subgroups within the dataset based on participants’ responses to 11 survey items related to general attitudes towards vaccines and immunization. LCA is a statistical technique used to identify unobserved subgroups within a population by analyzing patterns in observed data. LCA estimated the probability of each participant belonging to each subgroup and iteratively refined these probabilities using the Expectation-Maximization (EM) algorithm to reveal distinct underlying patterns [29]. Through this analysis, we classified the participants into two subgroups: one with vaccine-supportive attitude, and another with vaccine-concerned attitude.

Second, we created dichotomous variables for COVID-19 vaccine hesitancy or refusal. Participants who expressed willingness to receive or who had already received a COVID-19 vaccine were classified as the non-hesitancy group. Conversely, participants who had neither received a COVID-19 vaccine nor were willing to get one in the future were classified as the vaccine hesitancy group. The same categorization was applied to parents’ hesitancy or refusal regarding their children’s COVID-19 vaccination. For households with two or more children, if the participant reported hesitation to vaccinate at least one child, they were placed in the vaccine hesitancy group.

### 2.5. Statistical Analysis

We conducted bivariate analyses using weighted Chi-squared tests to examine the unadjusted associations between the terciles of the four social vulnerability domains and three types of attitude and behavior variables: (a) general vaccine attitudes, (b) adult COVID-19 vaccine hesitancy, and (c) parental vaccine hesitancy for children. Then, we performed weighted multivariable Poisson analyses with robust variance estimation for the three dependent variables to estimate the adjusted associations. We computed adjusted prevalence ratios (aPRs) and 95% confidence intervals (CIs). The models were adjusted for covariates including age (18–24, 25–34, 35–49, 50–64, and 65 or older), race and ethnicity (White, Hispanic, Black, Asian/Pacific Islander, and multi/other races), education (never attended school, elementary, some high school, high school graduate, some college or technical school, and college graduate or more), political affiliation (democrat, republican, independent, and something else), LGBTQ (LGBTQ and cisgender or heterosexual), marital status (married, divorced, widowed, separated, never married, and a member of an unmarried couple), dichotomized metropolitan status of their residential location, and data collection wave when the respondent participated. All data preparation and statistical analyses were performed using SAS Software (SAS Institute, version 9.4) and R (version 4.2.3).

## 3. Results

This study analyzed the data of 6716 participants in total, and the data of 2130 participants who have children. Overall, the unweighted sample had slightly fewer people aged 60 or older (12.8% vs. 15.3%), more Hispanic people (19.7% vs. 15.3%), more people with some college education or college graduates (67.8% vs. 61.0%), compared to the weighted sample (Table 2).

**Figure 1:**
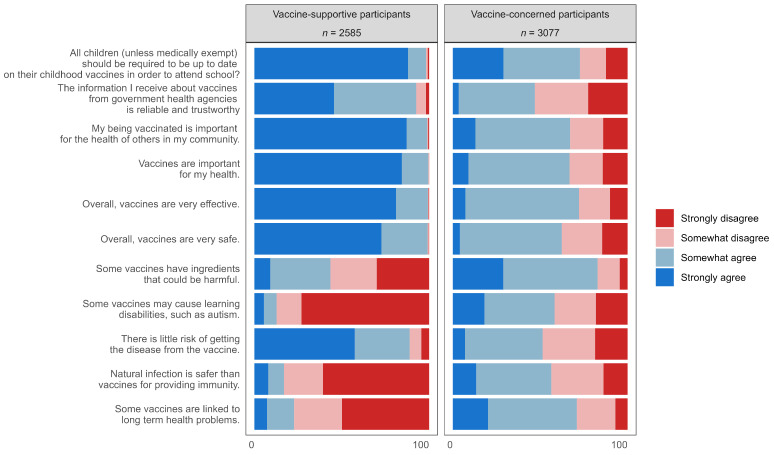
Identification of two Latent Classes of general vaccine attitudes and relative percentages of studied questions.

Figure 1 shows the response percentages for 11 survey items related to general vaccine attitudes and beliefs across the two subgroups identified by LCA. A total of 2585 participants were classified into the vaccine-supportive group, characterized by strong perceptions of vaccine safety, efficacy, and importance for both individual and community health, as well as a high agreement on the necessity of childhood vaccines. The remaining 3077 participants were classified into the vaccine-concerned group, characterized by a stronger perception of vaccine risk and less trust in the information they receive about vaccines from government health agencies.

**Figure 2:**
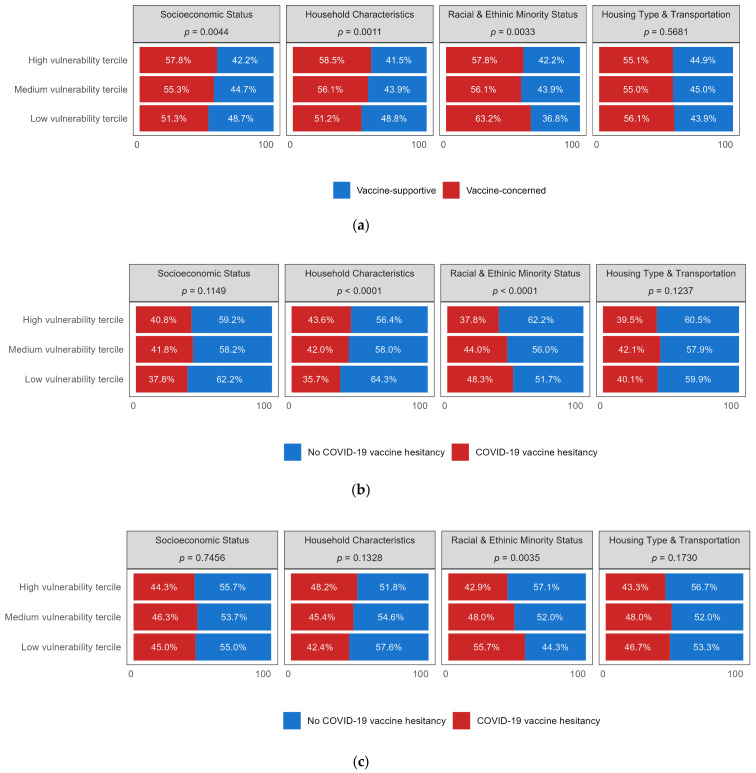
(**a**) General attitude toward vaccine by social vulnerability domain tercile; (**b**) adult COVID-19 vaccine hesitancy by social vulnerability domain tercile; (**c**) parental COVID-19 vaccine hesitancy for children by social vulnerability domain tercile.

The bivariate analyses depicted in Figure 2a show a significant association in three domains. The percentages of vaccine-concerned participants were significantly higher in counties in the high vulnerability tercile for the SES and Household Characteristics domains. Conversely, a significantly lower percentage of vaccine-concerned participants was observed in high vulnerability tercile counties for the Racial and Ethnic Minority domain.

Figure 2b displays the associations for the COVID-19 specific vaccine hesitancy. It shows that a higher percentage of participants had not received a COVID-19 vaccine or expressed hesitancy to get one in counties with higher Household Characteristics vulnerability. Conversely, lower percentages of participants had not received a COVID-19 vaccine or expressed hesitancy in counties with high Racial and Ethnic Minority vulnerability, compared to those in low vulnerability counties. A similar pattern was observed for parental COVID-19 vaccination hesitancy for children in this domain (Figure 2c): a higher percentage of participants did not have their children vaccinated or were not willing to do so in low vulnerability tercile counties (55.7%), compared to high vulnerability tercile counties (42.9%).

In the multivariable analyses (Table 3), for general vaccine attitude, living in higher vulnerability tercile counties was significantly associated with being less supportive of vaccine for the SES vulnerability domain (aPR 1.10; 95% CI 1.05–1.14). For COVID-19-specific vaccine hesitancy, residing in counties with a high vulnerability tercile for Household Characteristics domain was also significantly associated with not receiving COVID-19 vaccines or being unwilling to get one, compared to low tercile counties (aPR 1.13; 95% CI 1.07–1.20). Being consistent with the bivariate analysis, participants living in counties with high vulnerability tercile for Racial and Ethnic Minority domain were more likely to be positive about vaccines (aPR 0.91; 95% CI 0.88–0.94) and less likely to be hesitant toward the COVID-19 vaccine for themselves (aPR 0.81; 95% CI 0.76–0.87) and their children (aPR 0.84; 95% CI 0.75–0.94), compared to the low vulnerability tercile. Similarly, living in high vulnerability tercile counties for Housing Type and Transportation was significantly associated with having supportive attitude toward vaccine (aPR 0.92; 95% CI 0.89–0.95).

Multivariable Poisson analyses with robust variance estimation were conducted to estimate the prevalence ratios. The models were adjusted for key sociodemographic characteristics including age, race and ethnicity, education, political affiliation, marital status, metropolitan status of living location, and data collection wave where the respondent participated.

## 4. Discussion

Our study demonstrated that living in counties with high vulnerability related to SES and Household Characteristics domains was associated with being less supportive to vaccines and higher hesitancy toward adult COVID-19 vaccination. However, we observed different associations for the Racial and Ethnic Minority vulnerability and Housing Type and Transportation domains: residing in high vulnerability counties related to Racial and Ethnic Minority domain was associated with being more supportive to vaccines generally and lower hesitancy to COVID-19 vaccination for both adults and children, and residing in high vulnerability counties related to Housing Type and Transportation domain was associated with having more supportive vaccine-related attitudes, after adjusting for sociodemographic characteristics.

The positive associations between higher county-level vulnerability related to SES and Household Characteristics, and higher prevalence of vaccine concerns and hesitancy in our study, are consistent with prior research. Our study found larger proportions of populations living below 150% of the federal poverty line, people without health insurance, and disabled people were the strongest contributing factors to the higher rates of vaccine concerns and hesitancy in counties with greater vulnerability for these two domains (Supplementary Figure S1). Consistently, prior studies have reported higher rates of concerns about COVID-19 vaccine safety and unwillingness to get vaccinated among individuals with lower education, lower income, lack of insurance, and disabilities or immunocompromised conditions [30,31,32,33]. Although there is a limited body of literature analyzing disparities specifically related to general vaccine attitudes, lower SES was associated with other preventive measures as well, such as wearing a mask [34] and social distancing [35]. Several factors may explain these associations observed in our study. First, the most frequently suggested mechanism is that individuals with higher SES, such as higher income and better access to healthcare, typically have better access to healthcare information and a more accurate understanding of vaccine science, leading to a more favorable perception of vaccine safety and benefits [36,37]. Second, several prior studies suggested that individuals with higher vulnerability are more likely to have experienced historical inequalities within the healthcare system, which can undermine their trust in both the healthcare system and government institutions [38,39].

Parental COVID-19 vaccine hesitancy for children was not significantly associated with these two vulnerability domains in our study. A few prior studies have suggested that vaccine-hesitant parents in the U.S. were characterized by lower income and education levels [40]. In contrast, our study found no significant differences in parental vaccine hesitancy across any SES-related vulnerability components, including living under 150% of the federal poverty line and lower education level (Supplementary Figure S1). Concerns about vaccine side effects and parents’ perceptions of their children’s vulnerability to these side effects may have played a more significant role in parents’ pediatric COVID-19 vaccine hesitancy [25,41], potentially attenuating its association with the community-level vulnerability. This may explain the higher overall parental hesitancy rates compared to adults, but without distinct differences between county vulnerability tercile levels. Additionally, parents of children in different age groups may exhibit varying decision-making patterns regarding child COVID-19 vaccination. Analyses stratified by child age group are needed to better estimate age-specific associations.

The adverse associations observed in this study between higher Racial and Ethnic Minority vulnerability and lower COVID-19 vaccine hesitancy contrast with previous literature analyzing individual-level race and ethnicity data, which often found greater vaccine hesitancy among racial and ethnic minority groups, such as Black/African American and Hispanic individuals [18,42]. However, comparison with the previous studies analyzing individual-level racial and ethnic data should be approached with caution. Our findings are consistent to prior studies that examined county-level social vulnerability and actual vaccination coverage, which found higher COVID-19 vaccine coverage in counties with greater vulnerability related to Racial and Ethnic Minority status, as well as Housing Type and Transportation [43]. Several prior studies have documented similar disparities in other preventive measures: racial and ethnic minority groups were more likely to wear masks [44], and counties with higher proportions of limited-English speakers and more multi-unit housing were associated with higher rates of stay-at-home behavior [35]. Our results may reflect temporal changes due to governmental efforts that addressed initial COVID-19 vaccine hesitancy and improved vaccine perceptions through targeted interventions in communities with higher vulnerability scores for these two domains, Racial and Ethnic Minority Status and Housing Type and Transportation [45]. Prior evidence showed that the disparities in COVID-19 vaccination coverage among racial and ethnic minority groups narrowed during December 2020–November 2021 [46]. Another study supported this by suggesting that COVID-19 vaccine hesitancy decreased more rapidly among Black individuals than among White individuals [47]. Additionally, higher community-level COVID-19 incidence may have led to more self-protective health behavior in the socially vulnerable communities [48]. For instance, the experiences of disproportionate COVID-19 outcomes among racial and ethnic minority groups during early pandemic [49] may have increased the perceived importance of vaccines and reduced vaccine hesitancy in communities with higher proportions of racial and ethnic minority population. Third, counties with larger racial and ethnic minority populations or greater vulnerability characteristics related to the Housing Type and Transportation domain (e.g., housing in structures with 10 or more units) may correlate with more urban areas, where vaccine-related concerns and COVID-19 vaccine hesitancy were significantly less prevalent than rural areas (Supplementary Figure S1). Our analysis adjusted for the dichotomous urban/rural status of the county of residence, but it is possible that the effects of the continuous urbanicity continuum were not fully captured by the dichotomous measure.

### Limitations

This study has a few limitations. First, it utilized a sample from a non-probability panel, which can potentially lead to results that may not accurately represent the broader population due to the possibility of under- or over-representation of certain groups. However, research indicates that non-probability samples are becoming more accepted due to factors like limited budgets, high data collection costs, or urgency, if appropriate adjustments are made and limitations are clearly communicated [50]. While concerns about potential bias and its impact on representativeness remain, we found that the key demographic characteristics of the unweighted sample were reasonably close to those of the U.S. adult population. Furthermore, this limitation is less significant given that the primary aim of our research was to explore the relationship between county-level social vulnerability and vaccine-related attitudes and behavior, rather than to provide precise population point estimates. Second, as the COVID-19 vaccine became available from mid-April 2021, we asked vaccine hesitancy questions only to participants who had not received any doses of the vaccine. Participants who had already been vaccinated were classified into the non-vaccine hesitancy group for the final two data collection waves. While this approach provides a straightforward categorization of behavior intent based on actual behavior, it may introduce measurement errors. Specifically, this classification assumes that receiving the vaccine necessarily reflects an absence of hesitancy, which may not always be accurate. Some individuals might have been vaccinated despite holding hesitations or reluctance, possibly due to external pressures, mandates, or practical necessities related to work, school, and travel [51]. Despite this limitation, a strong correlation has been documented between lower vaccine hesitancy and a higher likelihood of getting vaccinated [52].

## 5. Conclusions

Our analyses underscore the ongoing significance of considering communities’ vulnerabilities related to socioeconomic status and household characteristics to mitigate disparities in vaccine-related perceptions and COVID-19 vaccine hesitancy within socially vulnerable populations. This highlights the importance of using the social vulnerability scores during public health emergencies to identify and support communities most likely to be disproportionately affected during future outbreaks. Our county-level evidence provides a foundation for developing more targeted and actionable area-based public health interventions, such as efficient and equitable resource allocation, compared to individual-level analyses. However, our study also demonstrated that the relationship between social vulnerability and vaccine-related attitudes, as well as COVID-19 vaccine hesitancy, varies across different components of social vulnerability. The multifaceted nature of social vulnerability and the varying patterns among its components underscore the need to account for these differential associations across the vulnerability components when designing more equitable and targeted community-based public health interventions to address disparities in vaccine hesitancy and enhance vaccine coverage.

## Figures and Tables

**Table 1:** Social factors used to evaluate overall social vulnerability score and four social vulnerability component domains.

Social Vulnerability	Socioeconomic Status	People below 150% federal poverty level
		People (age 16+) unemployed
		Housing cost burden
		People (age 25+) without high school diploma
		Noninstitutionalized people without health insurance
	Household Characteristic	People aged 65 and older
		People aged 17 and younger
		Noninstitutionalized population with a disability
		Single-parent households with children under 18
		People who speak English “less than well”
	Racial and Ethnic Minority Status	People of minority race groups, including: - Hispanic or Latino (of any race) - Black and African American, Not Hispanic or Latino - American Indian and Alaska Native, Not Hispanic or Latino - Asian, Not Hispanic or Latino - Native Hawaiian and Other Pacific Islander, Not Hispanic or Latino - Two or More Races, Not Hispanic or Latino - Other Races, Not Hispanic or Latino
	Housing Type and Transportation	Housing in structures with 10 or more units
		Mobile homes
		Housing units with more people than rooms
		Households with no vehicle available
		People in group quarters

**Table 2:** Sociodemographic characteristics.

	All Participants (*n* = 6716)			Participants with Children (*n* = 2130)		
	*n*	Unweighted %	Weighted %	*n*	Unweighted %	Weighted %
*Age*						
18–24	826	12.3	13.3	298	14.0	15.0
25–34	1312	19.5	18.9	520	24.4	24.4
35–49	1940	28.9	27.7	905	42.5	41.3
50–64	1781	26.5	24.8	330	15.5	14.9
65 or older	857	12.8	15.3	77	3.6	4.4
*Gender*						
Male	2995	46.1	48.2	960	45.1	47.2
Female	3705	53.7	51.6	1166	54.7	52.6
Missing	16	0.2	0.3	4	0.2	0.2
*Race/Ethnicity*						
white	4177	62.2	62.9	1178	55.3	56.5
Hispanic	1323	19.7	15.3	553	26.0	20.8
Black	581	8.7	11.8	193	9.1	12.2
American Indian/Alaska Native	45	0.7	0.7	10	0.5	0.5
Asian/Pacific Islander	268	4.0	4.4	87	4.1	4.6
Multi/Other races	275	4.1	4.2	100	4.7	5.0
Missing	47	0.7	0.7	9	0.4	0.5
*Education*						
Never attended school or only attended kindergarten	54	0.8	1.0	30	1.4	1.8
Grades 1 through 8 (Elementary)	63	0.9	1.0	27	1.3	1.6
Grades 9 through 11 (Some high school)	294	4.4	5.1	108	5.1	5.7
Grade 12 or GED (High school graduate)	1717	25.6	31.3	553	26.0	31.2
College 1 year to 3 years (Some college or technical school)	2348	35.0	30.9	728	34.2	29.9
College 4 years or more (College graduate)	2200	32.8	30.1	672	31.6	29.1
Prefer not to answer	40	0.6	0.7	12	0.6	0.6
*Income*						
Less than USD 25,000	1278	19.0	20.3	348	16.3	17.4
USD 25,000 to USD 34,999	923	13.7	14.3	282	13.2	13.8
USD 35,000 to USD 49,999	962	14.3	14.4	311	14.6	14.8
USD 50,000 to USD 74,999	1265	18.8	18.3	411	19.3	18.7
USD 75,000 to USD 99,999	884	13.2	12.9	317	14.9	14.8
USD 100,000 or more	1087	16.2	15.0	389	18.3	17.2
Do not know/Not sure	106	1.6	1.7	26	1.2	1.3
Prefer not to answer	211	3.1	3.2	46	2.2	2.1
*Political Party Identification*						
Democrat	2306	34.3	34.5	710	33.3	32.7
Republican	1942	28.9	28.7	610	28.6	28.4
Independent	1527	22.7	22.9	490	23.0	23.9
Something else	436	6.5	6.5	151	7.1	7.2
Prefer not to answer	505	7.5	7.5	169	7.9	7.8
*Sexual Orientation and Gender Identity*						
Lesbian, gay, bisexual, transgender, or queer	643	9.6	9.5	192	9.0	9.1
Cisgender and heterosexual	5905	87.9	87.9	1895	89.0	88.7
Do not Know/Not sure	96	1.4	1.5	28	1.3	1.5
Prefer not to answer	72	1.1	1.1	15	0.7	0.7
*Marital Status*						
Married	3067	45.7	44.0	1194	56.1	54.2
Divorced	777	11.6	11.5	177	8.3	8.3
Widowed	269	4.0	4.3	40	1.9	2.0
Separated	176	2.6	2.6	73	3.4	3.2
Never married	1760	26.2	27.3	446	20.9	22.4
A member of an unmarried couple	600	8.9	9.1	181	8.5	8.8
Prefer not to answer	67	1.0	1.1	19	0.9	1.0

**Table 3:** Association between Social Vulnerability terciles and vaccine-related attitudes and hesitancy.

	General Vaccine-Concerned Attitude	Adult COVID-19 Vaccine Hesitancy	Parental COVID-19 Vaccine Hesitancy for Children
	aPR (95% CI)	aPR (95% CI)	aPR (95% CI)
Socioeconomic Status			
Low tercile	1.00	1.00	1.00
Medium tercile	1.09 (1.05, 1.13) **	1.06 (1.00, 1.12) *	1.03 (0.94, 1.12)
High tercile	1.10 (1.05, 1.14) **	1.03 (0.96, 1.10)	0.99 (0.89, 1.10)
Household Characteristics			
Low tercile	1.00	1.00	1.00
Medium tercile	1.03 (1.00, 1.06) *	1.11 (1.06, 1.17) **	1.01 (0.94, 1.09)
High tercile	1.02 (0.99, 1.06)	1.13 (1.07, 1.20) **	1.07 (0.98, 1.17)
Racial and Ethnic Minority Status			
Low tercile	1.00	1.00	1.00
Medium tercile	0.92 (0.88, 0.95) **	0.93 (0.87, 0.99) *	0.90 (0.82, 0.99) *
High tercile	0.91 (0.88, 0.94) **	0.81 (0.76, 0.87) **	0.84 (0.75, 0.94) *
Housing Type and Transportation			
Low tercile	1.00	1.00	1.00
Medium tercile	0.93 (0.90, 0.96) **	1.04 (0.98, 1.10)	1.00 (0.93, 1.09)
High tercile	0.92 (0.89, 0.95) **	1.05 (0.99, 1.11)	1.00 (0.92, 1.08)

Abbreviations: aPR, adjusted prevalence ratio; CI, confidence interval, * *p* < 0.05, ** *p* < 0.001.

## Data Availability

The data presented in this study are available on request from the corresponding author due to commercial sensitivity.

## Supplementary Materials

The following supporting information can be downloaded at: https://www.mdpi.com/article/10.3390/vaccines12121368/s1, Table S1: Panel survey sample sizes and dates by wave; Figure S1: Percentages of vaccine-concerned attitudes, adult COVID-19 vaccine hesitancy, and parental COVID-19 vaccine hesitancy for children by individual social vulnerability scores and rural/urban status.
