# Supplementary material for: Association Between County-Level Social Vulnerability and Vaccine-Related Attitudes and Hesitancy Toward COVID-19 Vaccination in the United States
Source: Vaccines (Basel). 2024 Dec 3;12(12):1368. doi: 10.3390/vaccines12121368 (PMC11680208; doi:10.3390/vaccines12121368)
Supplement: Supplementary file 1 [file vaccines-12-01368-s001.zip › vaccines-3281293-supplementary.pdf]

vaccines

MDPI

Supplemental Material

Supplementary Table S1. Panel survey sample sizes and dates by wave.

Wave	Fielding Period	Sample Size
1	7/23/2020–7/29/2020	931
2	8/24/2020–8/31/2020	974
3	9/23/2020–10/04/2020	974
4	10/26/2020–11/01/2020	920
5	11/18/2020–12/01/2020	917
6	4/15/2021–4/21/2021	1000
7	8/19/2021–8/31/2021	1000

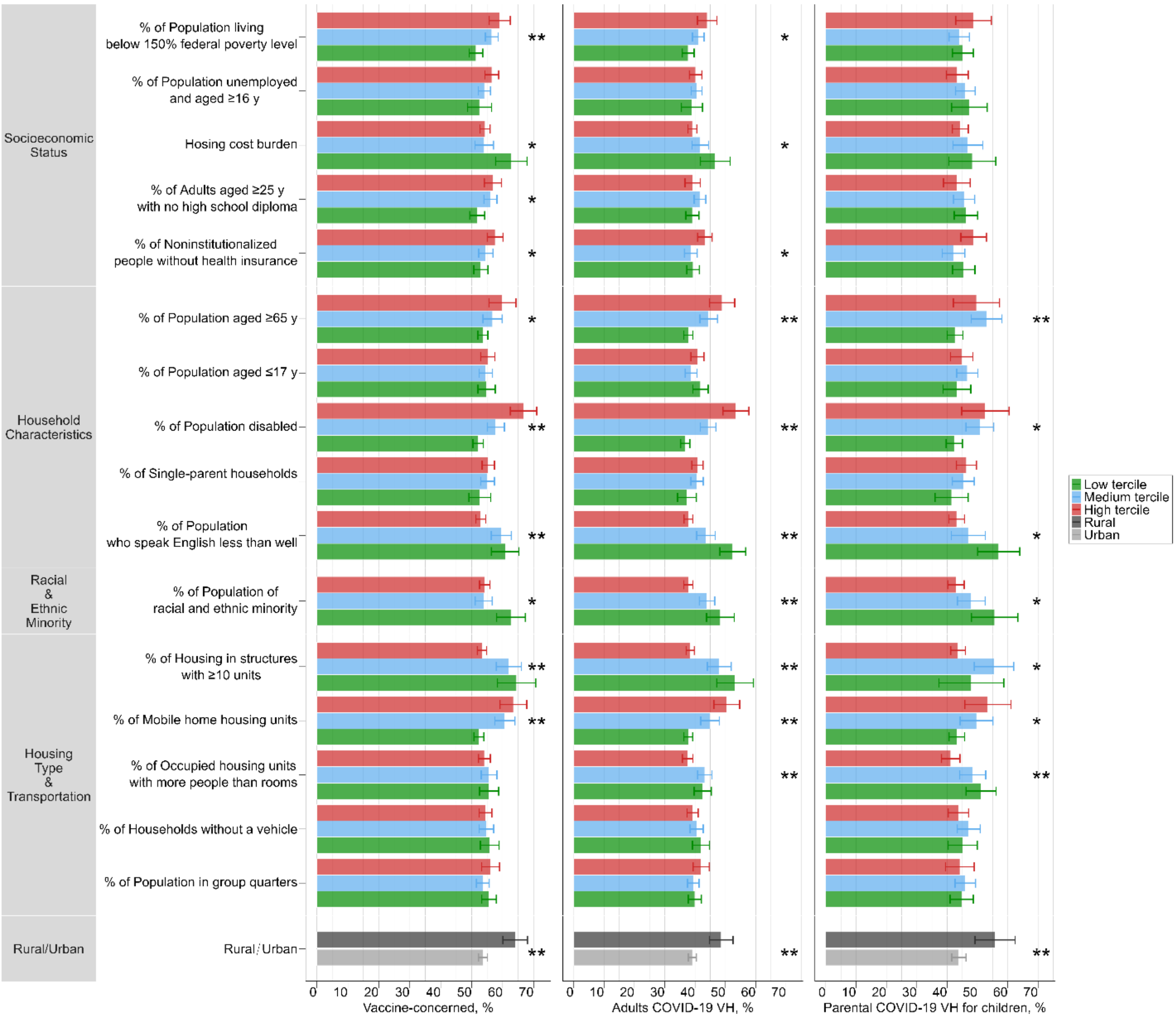

Supplementary Figure S1. Percentages of vaccine-concerned attitudes, adults COVID-19 vaccine hesitancy, and parental COVID-19 vaccine hesitancy for children by individual social vulnerability scores and rural/urban status (weighted). Abbreviations: VH Vaccine Hesitancy, * $p < 0.05$, ** $p < 0.001$.
